# A Bivalent MAPS Vaccine Induces Protective Antibody Responses against *Salmonella* Typhi and Paratyphi A

**DOI:** 10.3390/vaccines11010091

**Published:** 2022-12-30

**Authors:** Fan Zhang, Emily M. Boerth, Joyce Gong, Nicole Ma, Katherine Lucas, Olivia Ledue, Richard Malley, Ying-Jie Lu

**Affiliations:** Division of Infectious Diseases, Boston Children’s Hospital, Harvard Medical School, 300 Longwood Avenue, Boston, MA 02115, USA

**Keywords:** *Salmonella*, bivalent vaccine, Vi, OSP

## Abstract

Infections by Salmonella Typhi and Paratyphi A strain are still a major cause of morbidity and mortality in developing countries. Generation of antibodies against the Vi capsular polysaccharide of *S*. Typhi via either pure polysaccharide or protein–polysaccharide conjugate is a very effective way to protect against *S*. Typhi. To date, there is no commercially available vaccine against *S*. Paratyphi A. The O-specific polysaccharide (OSP) has been generally considered a good vaccine target for Paratyphi A. Here, a bivalent vaccine against Vi and OSP was generated using the Multiple Antigen Presenting System (MAPS). Three different protein constructs, including CRM197, rEPA of Pseudomonas, and a pneumococcal fusion protein SP1500-SP0785, were fused to Rhizavidin (Rhavi) and evaluated their impact on immunogenicity when incorporated as fusion proteins affinity-bound to the two polysaccharides. We compared the antibody responses, antibody avidity, and cidal activity of sera post-immunization with monovalent vs. combination vaccines. We also wished to evaluate the generation of Vi-specific memory B cells in mice. We found little interference when combination vaccine was compared to monovalent vaccines with respect to antibody concentration and cidal activity of sera. Significant affinity maturation was noted for both Vi and OSP antigens. Thus, our preclinical results with a combination Vi- and OSP-MAPS vaccine strongly support the feasibility of this approach and its application of this approach to other important salmonella and Shigella species.

## 1. Introduction

Enteric fever, caused by several Salmonella enterica subspecies, remains an important cause of morbidity and mortality in developing countries. It is estimated that *Salmonella* Typhi and Paratyphi A caused 14.3 million cases of typhoid and paratyphoid fevers and 1359 thousand deaths globally in 2017 [1]. Additionally, the emergence of resistance to commonly used antimicrobials in *S*. Typhi has dramatically reduced the options for clinical management, and there is an urgent need to develop effective vaccines against these enteric diseases [2,3].

Surface polysaccharides of many bacteria have been used as effective antigens, such as capsular polysaccharides or associated with lipopolysaccharide (LPS) as in the case of Gram-negative enteric bacteria [4]. The Vi capsular polysaccharide of *S.* Typhi is an important virulence factor and a protective antigen [5,6,7]. Vi is a linear polymer composed of (α1-4)-2-deoxy-2-*N*-acetyl galacturonic acid moieties and is a thymus-independent antigen. Immunization with Vi conjugated to the nontoxic recombinant exotoxin A of *Pseudomonas aeruginosa* (Vi-rEPA) was shown to elicit high levels of serum anti-Vi IgG in infants and young children [8,9], following which two Vi-protein conjugates using either tetanus toxoid (TT) [10] or CRM197 [11] as carrier proteins were licensed. A recent analysis of typhoid conjugate Typbar-TCV vaccination in India showed that vaccination could reduce mortality and was highly cost-effective [12]. Several follow-up clinical trials supported by the Typhoid Vaccine Acceleration Consortium evaluated the post-licensure efficacy and effectiveness of Typbar in three countries. They found that the vaccine efficacy against culture-confirmed typhoid fever was approximately 80% at 18–24 months in all study sites [13,14,15]. In addition, a Vi-Dt (where Dt stands for Diphtheria toxoid) conjugate vaccine was proven to be safe, immunogenic, and non-inferior to the Vi-TT vaccine at 4 weeks post-vaccination of a single dose [16].

In contrast, paratyphoid vaccine development is lagging. Currently, no vaccine against *S*. Paratyphi A is available despite a possible increase in the incidence of enteric fever caused by *S*. Paratyphi A [17]. *S*. Paratyphi A O-specific polysaccharide (OSP)-protein conjugates were previously shown to be safe and to elicit anti-OSP IgG antibodies in different age groups [18,19]. Other approaches have included live attenuated strains [20,21] or OSP conjugates made with other carriers [22,23], which have been evaluated in preclinical models.

The Multiple Antigen Presenting System (MAPS) is a vaccine platform that uses the affinity pair biotin-rhizavidin to generate a complex of polysaccharides and proteins [24,25,26,27] that can generate antibodies against polysaccharides and proteins. MAPS-based vaccines induce robust, boostable and CD4+ T cell-dependent anti-polysaccharide antibody responses, as well as functional antibodies and Th1/Th17cell response to carrier proteins, which may provide additional benefits over conventional conjugate technology. A 24-valent pneumococcal MAPS vaccine has completed a Phase 2 trial in older adults and is currently undergoing a Phase 2 trial in infants. In the current study, we applied MAPS technology to make a bivalent vaccine with Vi and Paratyphi A OSP. Here, we show the generation of memory B cells and affinity maturation by the MAPS vaccine, which characterizes the immune response to traditional conjugate vaccines. We further demonstrate that a combination of Vi and OSP MAPS vaccine generated long-lasting, boostable, and bactericidal immune responses in rabbits. Our studies thus strongly support the feasibility of this approach and the application of this approach to not only these two important targets but also other diarrheal pathogens.

## 2. Materials and Methods

Materials: Aluminum phosphate (alum) was from Brenntag North America (Reading, PA, USA). Vi polysaccharide was provided as a gift by Dr. Szu from NIH [28]. GMP-grade Vi polysaccharide used in the GLP toxicology study was purchased from Walvax, China (Yuxi, Yunnan). A Vi-Dt conjugate vaccine was kindly provided as a gift by Dr. Carbis from International Vaccine Institute. Adipic acid dihydrazide (ADH), 1-Ethyl-3-[3-dimethylaminopropyl] carbodiimide Hydrochloride (EDC) and N-hydroxysulfosuccinimide (NHS) were purchased from Thermofisher (Waltham, MA, USA). Restriction endonucleases and T7 shuffle express competent cells were purchased from New England Biolabs (Ipswich, MA, USA). All other reagents were obtained from Sigma (St Louis, MO, USA).

Protein Purification. DNA fragments encoding SP1500-SP0785, CRM197, and rEPA were cloned into a pET21b vector containing Rhazavidin (Rhavi) by restriction enzyme digestion and ligation. Sequence-confirmed plasmids were transformed into *E. coli* T7 shuffle express cells and transformants containing the relevant cloned proteins were grown to OD600 = 1 at 25 °C. Protein expression was induced with 0.2 mM IPTG at 16 °C overnight. Cells were spun down, and pellets were resuspended in lysis buffer (20 mM Tris-HCl, 500 mM NaCl, pH8.0) and then lysed by sonication using a probe sonicator with 30 s sonication and rest 30 s resting (on ice). The proteins of interest were purified from the supernatant over a Ni-NTA column; proteins were eluted in imidazole buffer. Protein-containing elutions were combined and purified over a gel-filtration column for dimer fractions.

Purification of OSP from *S*. Paratyphi. *S*. Paratyphi A strain 9150 (ATCC) was modified as described previously with some modifications [29]. The *Wzz* gene was deleted with a suicide pCACTUS plasmid carrying *S*. Paratyphi SPA0792 (*wzzB*) gene. The resulting mutant was transformed with a pBluescript plasmid carrying the *S*. Paratyphi *fepe* gene under the control of the promoter of pTac from *S*. Paratyphi. The resulting strain (YL133) produces a higher molecular weight LPS. OSP was purified from YL133 using a protocol established previously [30]. Briefly, cells were resuspended in 2% acetic acid and boiled at 100 °C for 2 h. Ammonium hydroxide was added to neutralize the pH, and the pellet was removed by centrifugation. The supernatant was dialyzed against water three times before adding citrate buffer to a final concentration of 20 mM (pH3). Protein precipitation was removed by centrifugation. Buffers were added to reach a final concentration of 18 mM Na2PO4, 24% ethanol and 200 mM CaCl2. The solution was incubated at 4 °C overnight before centrifugation to remove nucleic acids. The supernatant was dialyzed against water three times before lyophilization. The size of OSP was further increased by linking the terminal aldehyde group using adipic acid dihydrazide (ADH) as a linker. OSP at 10–20 mg per ml was mixed with ADH in PBS (100 mg/mL) at a weight ratio of 1:1. The reaction was kept at room temperature, stirring for 2 h. The resulting ADH-OSP was dialyzed extensively with PBS and mixed with OSP at a concentration of 10 mg/mL in the presence of 50 mM sodium cyanoborohydride in PBS, stirred at RT overnight. Final OSP was dialyzed against water extensively before being frozen at −80 °C.

Biotinylation of Vi and OSP. Vi was biotinylated with Amine-PEG3-Biotin as described previously with minor modification [27]. Briefly, Vi was resuspended to 5 mg/mL in buffer A (0.2 M MES, 150 mM NaCl, pH 5.8), EDC (100 mg/mL in buffer A), and NHS (100 mg/mL in Dimethylformamide) were added into the solution for 15 min at room temperature. The pH was adjusted to 7.0 by adding 1M NaHCO_3_ (pH 10). Amine-PEG3-Biotin (40 mg/mL in water) was added to a ratio of 1:1 (*w*:*w*). The reaction was stirred for another 2 h at RT before adding glycine to 20 mM final concentration. OSP was biotinylated with CDAP (1-cyano-4-dimethylaminopyridinium tetrafluoroborate) using the protocol described previously [27,31]. Biotinylated Vi and OSP were dialyzed against saline extensively before being used for MAPS assembly. MAPS was assembled at a 3:1 (*w*:*w*) protein: polysaccharide ratio and purified with size exclusion columns. Protein concentration was determined by the BCA method (Pierce), and Vi concentration was determined by the acridine orange method [32]. OSP concentration was determined using the Anthrone method [33].

Antigen preparations and immunization. For immunization with Vi-MAPS alone, the vaccine was mixed with saline and injected directly into animals. For immunization experiments with OSP MAPS alone or bivalent vaccine, vaccines were mixed with aluminum phosphate (alum) at the indicated concentration in a 5 mL tube, which was then tumbled overnight at 4 °C to allow for adsorption one day before immunization. All mouse experiments, including subcutaneous immunizations and adoptive transfer, were performed at Boston Children’s Hospital. Guinea pig and rabbit intramuscular immunization experiments were carried out at Cocalico Biologicals Inc. All animal studies were approved by our local animal ethics committees or those at Cocalico Biologicals.

Adoptive transfer. WT C57BL/6J mice were immunized with either Dt-Vi conjugate [34] or rEPA-Vi MAPS once subcutaneously and rested for 6 weeks before being used as donor mice. Splenocytes were separated from donor mice and intravenously transferred to Rag^−/−^ mice (equivalent number of cells from one spleen per mouse). Rag^−/−^ mice were immunized 7 days post transfer with a mixture of 5 μg each of Vi, Dt and rEPA. Blood samples were obtained from Rag^−/−^ mice just before cell transfer and then again 14 days post-immunization, and serum antibody levels against Vi or proteins were analyzed by ELISA.

Enzyme-linked immunosorbent assay (ELISA). IgG antibody titers against Vi and OSP were measured using previously described methods [19,35]. Antibody avidity was measured by determining the concentration of sodium thiocyanate required to elute 50% of antibody from ELISA plates as published before with modifications [36]. Briefly, 50 μL of sera with 1:100 dilution in PBS/0.05% tween (PBST) was added to plates coated with Vi or OSP and incubated at room temperature for 30 min. Series dilutions of sodium thiocyanate were added to each well, and the plates were incubated at room temperature for another 2 h. Plates were washed, added HRP-secondary antibody in PBST and incubated at room temperature for 1 h. Plates were developed after washing with TMB substrate (Sureblue). The affinity index was defined as the concentration of sodium thiocyanate at which a 50% of antibody binding was achieved.

Bacterial killing assays. Salmonella killing assays were performed as described previously [35,37,38] using a Salmonella typhimurium strain carrying an empty vector (Strain C5) or expressing Vi polysaccharide on the surface (Strain C5.507) [35]. Briefly, bacteria were mixed with heat-inactivated antibodies for 20 min at room temperature. Differentiated HL-60 cells and baby rabbit complement were added in and the assay plates were shaken at 700 rpm at 37 °C for 1 h. The assay plates were placed on ice for 20 min before the addition of 1% saponin to lyse the cells. The samples were plated on blood agar plates to determine the remaining bacteria. Bactericidal activity for *S*. Paratyphi was carried out as described previously using ATCC 9150 strain [39]. Briefly, bacteria were mixed with heat-inactivated antibodies and baby rabbit complement. The assay plates were shaken at 700 rpm at 37 °C for 1 h. The samples were plated on blood agar plates to determine the remaining bacteria.

Toxicology Study. A bivalent Rhavi-SP1500-SP0785 (CP1)-Vi and CP1-OSP MAPS vaccine was produced under Good Laboratory Practice (GLP) and formulated by Vaxform Inc. A GLP-toxicology study was performed at IIT Research Institute (IITRI, Chicago, IL, USA). The vaccine was given at target doses of 25 μg of Vi antigen + 25 μg of Paratyphi OSP antigen + 250 μg of aluminum phosphate adjuvant on study days 1 and 15. Blood samples for immunogenicity analyses were collected at pre-test, before dosing on Study Day 15, and on Study Days 17 and 29. All other examinations were performed according to the protocols at IITRI.

Statistical analysis. Statistical analysis was carried out using the Mann–Whitney *U* test and PRISM (version 8.12, GraphPad Software, Inc., San Diego, CA, USA).

## 3. Results

### 3.1. Generation of Memory B Cell by MAPS Vaccine

**Figure 1:**
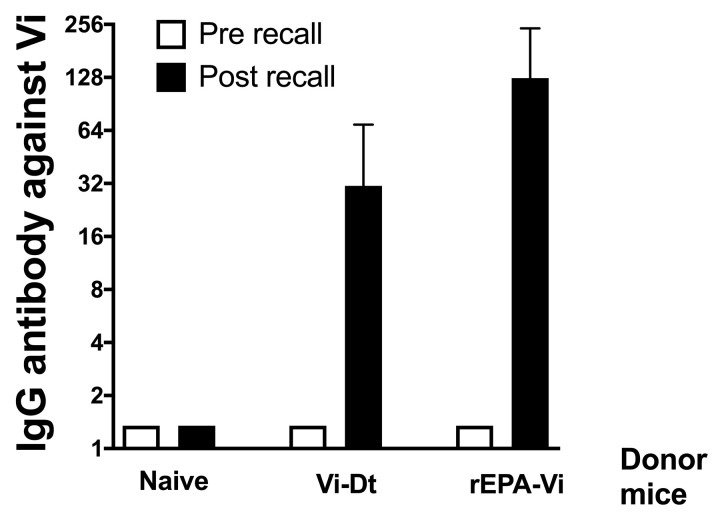
Recall of memory response for Vi-Dt conjugate and rEPA-Vi MAPS complex. The adoptive transfer was carried out as described in the “Materials and Methods” section. Each group contained five mice.

A hallmark of conjugate vaccines is the generation of T-dependent IgG-producing memory B cells [40]. As the MAPS platform does not rely on the chemical conjugation of protein and polysaccharide, it was important to test whether the Vi MAPS vaccine generates this type of response in the mouse model. rEPA was selected as a carrier protein because of the previous success of rEPA-Vi conjugate [41,42,43]. rEPA was fused to Rhavi and purified by Ni-NTA and FPLC. Vi MAPS were formed and purified from the superdex S500 column. A Dt-Vi conjugate vaccine was used as a positive control in this experiment. As shown in Figure 1, immunization of rEPA-Vi MAPS generated Vi-specific memory B cells: Rag^−/−^ mice who had received adoptive transfer of B and T cells from previously immunized wild-type C57BL/6 mice generated anti-Vi IgG upon recall with Vi polysaccharide. Rag^−/−^ mice that received splenocytes from naïve mice did not generate any Vi antibody, whereas mice that received Vi-Dt splenocytes behaved similarly to those that received the MAPS vaccine. As a control, immunization also generated significantly higher Dt- or rEPA-specific IgG antibodies in mice that received splenocytes from Dt-Vi or rEPA-Vi immunized mice, respectively.

### 3.2. Comparing Different Fusion Proteins in Vi-MAPS

**Figure 2:**
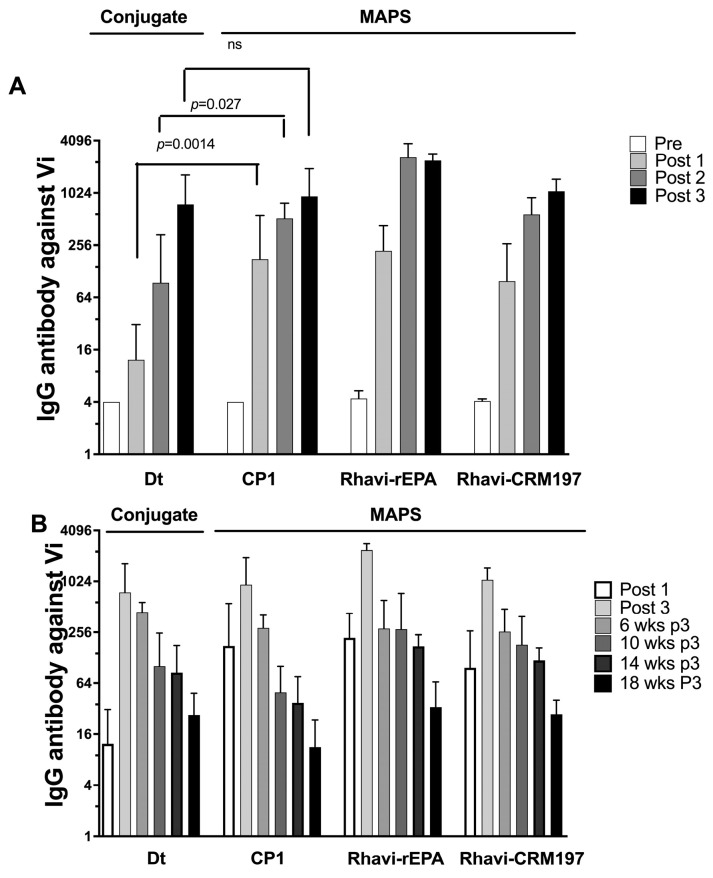
Immunogenicity of Vi conjugate or Vi MAPSs in guinea pigs. (**A**). Vi IgG titer from Dt-Vi conjugate, CP1-Vi, Rhavi-rEPA-Vi and Rhavi-CRM197 MAPS immunized Guinea pigs. (**B**). Vi IgG titer kinetics after the last immunization. Each group contained ten Guinea pigs. Bars showed geometric mean antibody titers and 95% confidence intervals. ns, non-significance.

Two additional proteins, including Rhavi-CRM197 and CP1 were evaluated as protein components of MAPS based on prior use and experience with other MAPS vaccines (Rhavi-SP1500-SP0785 has tested as a carrier protein for pneumococcal MAPS vaccine in phase one/two clinical trials) [23,44,45,46]. We evaluated the immunogenicity of these 3 MAPS constructs in rabbits as well as guinea pigs (GP), two species which, unlike mice, do not generate serum antibodies in response to injection of pure Vi [47]. We first evaluated the overall immune responses to these MAPS vaccines containing 5 μg of Vi in rabbits. Rabbits were immunized three times at a two-week intervals, and anti-Vi IgG was measured two weeks after the last immunization and subsequently every four weeks. As shown in Figure 2A, all three carrier proteins generated a Vi-IgG response with a titer increase after each immunization. CP1-Vi MAPS immunized GP had significantly higher Vi antibody post 1 and 2 immunizations than Vi-Dt MAPS. The two other fusion proteins showed a similar trend as CP1. Duration of Vi IgG antibody was then evaluated, given in these immunized GPs as a Vi-CRM197 conjugate vaccine had shown short antibody duration in a phase II clinical trial in children [48]. As shown in Figure 2B, all three MAPS vaccines had a similar rate of Vi antibody persistence, with a similar rate of decline. Thus, animal experiments in GPs suggested that the MAPS vaccine generated similar or higher responses to the Dt-Vi conjugate vaccine. The three carrier proteins showed similar immunogenicity in all the animal experiments; however, CP1 has a better purification profile compared to the other two proteins. In addition, CP1 was safe and immunogenic in recent human clinical trials. Thus, we selected CP1 as the fusion protein for future studies.

### 3.3. Purification of Paratyphi A OSP

Using a pneumococcal cell wall polysaccharide (CWPS), we previously demonstrated superior immunogenicity with larger polysaccharides [49]. We confirmed these findings with other polysaccharides (PS) (unpublished data). OSP purified from wild-type paratyphi A strain has a size of 40–50 kDa as previously reported [23]. We thus evaluated two strategies to increase the size of OSP by deleting the *wzzB* gene and overexpressing the *fepe* gene as described in Material and Methods. The purified OSP showed a molecular weight of 80 kDa by size-exclusion column-multiple angle laser light scattering (SEC-MALS).

### 3.4. Alum Phosphate Is Required for Antibody Production with OSP-MAPS

**Figure 3:**
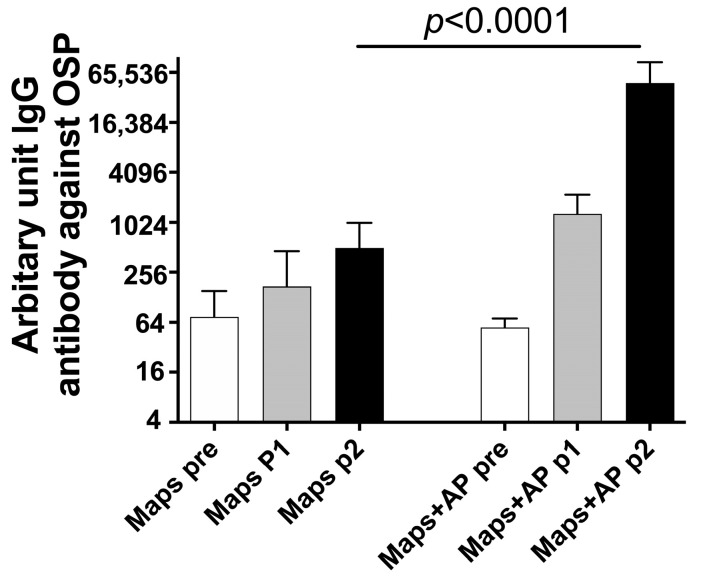
The effect of alum phosphate adjuvant on OSP antibody generation. Rabbits were immunized with CP1-OSP MAPS. Each group contained ten rabbits. Bars showed geometric mean antibody titers and 95% confidence intervals.

Rabbits were immunized with CP1-OSP MAPS with or without alum phosphate. As shown in Figure 3, after one and two immunizations, rabbits who received MAPS without AP had about a 2- and a 7-fold increase in anti-OSP IgG antibodies, respectively. In contrast, rabbits who received MAPS and AP had a 20- and 800-fold increase in anti-OSP IgG antibodies. Interestingly, this AP-dependent enhancement of antibody production was not observed with OSP conjugate or Vi-MAPS, pointing to an adjuvant effect specific for Paratyphi A OSP MAPS.

### 3.5. Immunogenicity of Bivalent MAPS in Rabbits

**Figure 4:**
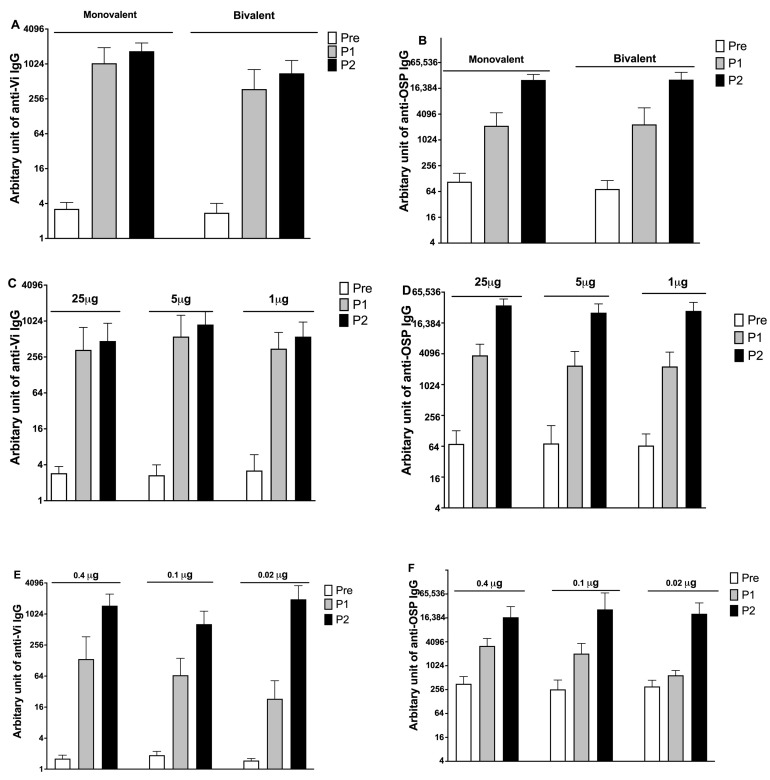
Immunogenicity of the monovalent and bivalent vaccines and dose responses for the bivalent vaccine. (**A**). Anti-Vi IgG titers for the monovalent and bivalent vaccines after one and two immunizations. (**B**). Anti-OSP IgG titers for the monovalent and bivalent vaccines after one and two immunizations. (**C**,**E**). Dose responses of Vi MAPS in rabbits. (**D**,**F**). Dose responses of OSP MAPS in rabbits. Each group contained ten rabbits. Bars showed geometric mean antibody titers and 95% confidence intervals.

We further tested whether immunization with a bivalent Vi and OSP MAPS can generate an immune response in rabbits. CP1-Vi and CP1-OSP MAPS were mixed at a 1:1 ratio (25 μg of each polysaccharide) in the presence of AP and then immunized rabbits. Sera were taken two weeks post-one or -two immunizations, and antibody titers were measured against Vi and OSP (Figure 4). Compared to the monovalent MAPS vaccine formulated at the same dose of polysaccharide, there was a small, but significant reduction in anti-Vi IgG titer after one (about 3-fold lower, *p* = 0.0355) and two immunizations (about 2-fold lower, *p* = 0.005) (Figure 4A). The anti-OSP IgG titer was not statistically different in rabbits who received monovalent or bivalent vaccines (Figure 4B).

We then tested dose responses of bivalent MAPS in rabbits. Three groups (1, 5, and 25 μg) were evaluated in rabbits. As shown in Figure 4C,D, there was no significant difference between the three doses for antibody titer against either Vi or OSP. Three more concentrations were tested in rabbits, as shown in Figure 4E,F. In these lower dose groups, antibody titers were lower after the first immunization in the rabbits who received a lower dose of vaccines. Nonetheless, antibody titers after the second immunization reached similar levels between different dose groups.

### 3.6. Antibody Duration and Affinity Maturation

**Figure 5:**
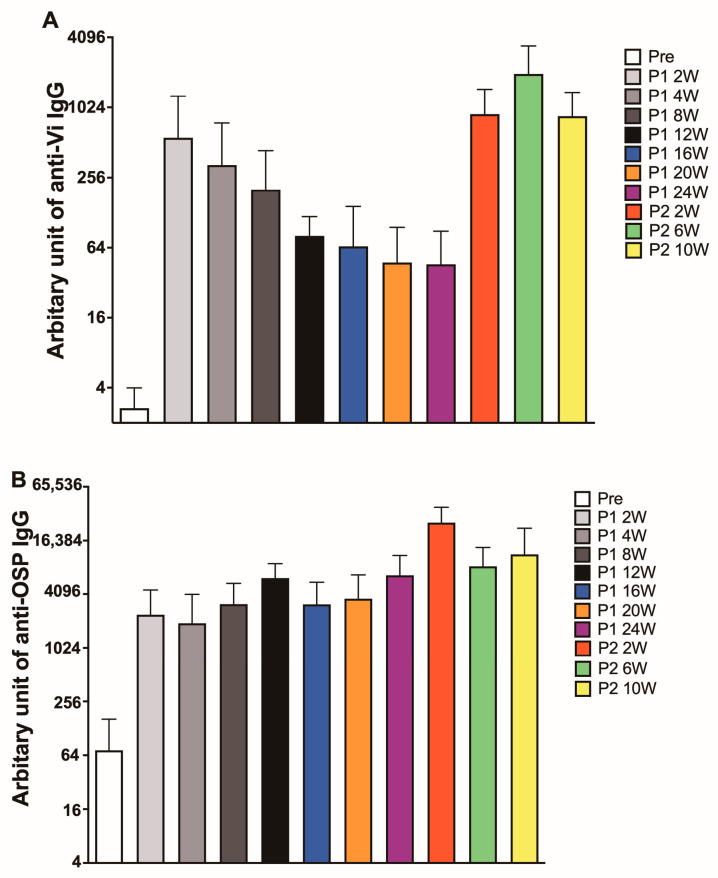
IgG kinetics in rabbits that received one or two immunizations of the bivalent vaccine. (**A**). Anti-Vi IgG titer kinetics. (**B**). Anti-OSP IgG titer kinetics. Each group contained ten rabbits. Bars showed geometric mean antibody titers and 95% confidence intervals.

Vi igG following immunization with a recently licensed Vi conjugate vaccine (Typbar-TCV, Bharat (Hyderabad, India)) persisted for up to two years in children [10]; subsequently, it was also shown that one dose of Typbar-TCV induced an anti-Vi antibody above baseline for five years [50]. However, an investigational Vi-CRM conjugate vaccine showed significant titer decline after 6 months of immunization in infants and did not show a boosting effect [48]. Thus, we decided to study the duration and the boosting effect of our bivalent vaccine in the rabbit model. As shown in Figure 5A, the titer of the Vi antibody started to decline at 4 weeks after the first immunization and continued to fall until a plateau at 12 weeks after immunization. The titer of Vi had an 18-fold boost after the second immunization, and the titer of Vi antibody stayed relatively the same for up to 10 weeks of follow-up.

**Figure 6:**
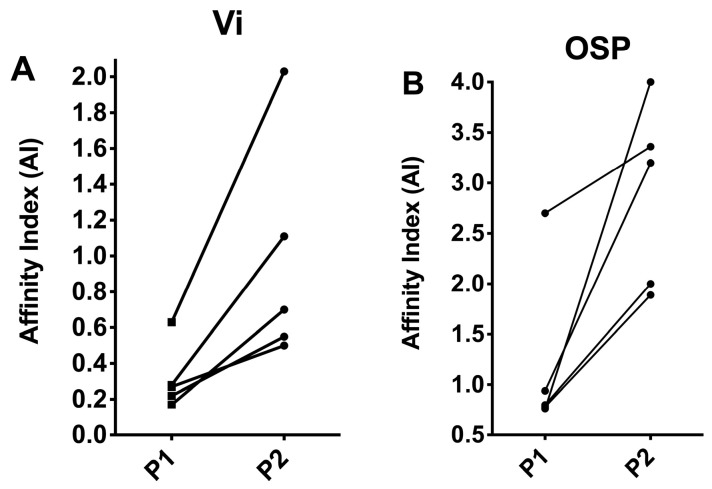
Affinity maturation of Vi (**A**) and OSP (**B**) antibodies. Affinity index was calculated as described in the “Materials and Methods” section.

In contrast, the titer of the OSP antibody stayed at the same level for 24 weeks after the first immunization and had a 4-fold rise after the second immunization. OSP antibody stabilized at the level of post-1 immunization at 6 or 10 weeks after the second immunization. We then compared the affinity of antibodies against Vi and OSP after one or two immunizations to determine whether there was affinity maturation in their antibody, a hallmark of conjugate vaccines. Thus, the matching pair of sera obtained in Figure 4 at 2 weeks after one or two immunizations were treated with different concentrations of sodium thiocyanate to determine their affinity indexes. As shown in Figure 6A,B, antibodies against Vi and OSP MAPS had increased affinity after the second immunization, suggesting that the second immunization induced affinity maturation for the MAPS vaccine.

### 3.7. Functional Assay of Rabbit Serum

**Figure 7:**
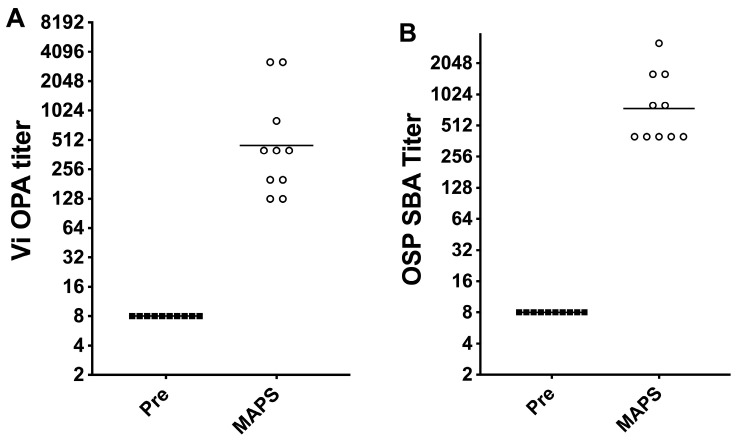
OPA titers of anti-Vi antibodies (**A**) and BCA titers of anti-OSP antibodies (**B**). Rabbits were immunized with the bivalent vaccine twice, and sera were taken two weeks after the last immunization.

We further analyzed the in vitro function of antibodies produced by bivalent MAPS two weeks after the second immunization. The killing activity of the Vi antibody was measured by an opsonophagocytic assay using heat-inactivated serum, baby rabbit complement, and differentiated HL60 cells with a Vi-expressing salmonella Typhimurium strain as described previously [35]. OPA titer of Vi antibody as defined by the dilution of serum to have at least 50% killing is shown in Figure 7A. The titers of post-immunization sera ranged from 64 to 1600, and no killing was detected in pre-serum. We also performed OSP serum bactericidal killing assay with baby rabbit complement and heat-inactivated serum against *S*. Paratyphi 9150 strain [39]. As shown in Figure 7B, the titers of post-immunization serum ranged from 200 to 1600, while pre-immunization serum had no measurable killing activity.

### 3.8. Toxicology Study

The bivalent MAPS was produced under Good Laboratory Practice (GLP) and tested in a rabbit toxicology study. Intramuscular administration of the bivalent vaccine was well tolerated. There were no treatment-related, toxicologically significant, or adverse effects seen for clinical observations, physical examinations, inoculation site reactogenicity, body weights, body weight changes, food consumption, body temperatures, ophthalmology, clinical chemistry, hematology, organ weights, and macroscopic pathology. Furthermore, treatment with the bivalent vaccine induced robust antibody production for the three components in the vaccine: OSP, Vi, and CP1, similar to what we have observed in previous rabbit immunogenicity studies.

## 4. Discussion

We have designed a bivalent vaccine using MAPS technology that targeted two important enteric pathogens: *S*. Typhi and *S*. Paratyphi, and tested its immunogenicity in various animal models. Our results showed that both Vi-MAPS and OSP-MAPS are immunogenic, and induce antibody affinity maturation and serum bactericidal activity. We also showed that the MAPS vaccine generated memory response in the mouse model and long-lasting circulating antibodies.

A carrier protein is essential for producing the polysaccharide antibodies of the conjugate vaccines [51]. CRM197 has been used in pneumococcal conjugate vaccine Prevnar and other conjugate vaccines as a carrier protein to induce antibody production against polysaccharides, including Vi-CRM197 and O2-CRM197 conjugate vaccines that have been tested in animal models and infants [23,44,48]. On the other hand, rEPA was never used in licensed vaccines but has been tested in many investigational vaccines in human trials [8,9,52,53,54]. All three fusion proteins studied here showed excellent immunogenicity in our animal models (Figure 2). While we did not identify important differences in immunogenicity between the three fusion proteins we evaluated, we selected CP1, based on ease of purification, and safety in human clinical trials [45,46]. The safety profile of our toxicology study was excellent, further supporting the selection of CP1.

We have recently shown that MAPS can induce pneumococcal polysaccharide-specific T and B memory cells. Here, we generated additional data showing that MAPS can induce polysaccharide-specific B memory cells. Unlike in a traditional conjugate vaccine, with MAPS, the connection between polysaccharide and carrier proteins is not a covalent bond; instead, the high-affinity binding between Rhavi and biotin is stable enough to facilitate the internalization of MAPS complex and presentation of polysaccharide through MHCII dependent pathway. We showed that Vi MAPS generated a higher level of IgG antibody than Vi conjugated to Dt after the first and second immunizations. The antibody level plateaued after three immunizations, and the difference between MAPS and conjugate was not significant (Figure 2). Our preclinical observations confirmed that MAPS generated similar immune responses as traditional conjugate vaccines.

Our study showed that the antibodies against Vi and OSP had different kinetics (Figure 5). The reason for this discrepancy is unknown. Vi antibody declined after its peak at 2 weeks, but its titer at week 24 was still 15 times higher than the baseline titer at pre-immunization. A similar decline of Vi antibody was also observed in GPs (Figure 2B). Antibody against OSP did not decline significantly during these 24 weeks. Even though a second immunization boosted the antibody titers for both PS, one immunization is sufficient to generate a long-lasting high antibody response for both Vi and OSP, at least in rabbit models.

## 5. Conclusions

We conclude that this bivalent Vi and paratyphi OSP MAPS vaccine is highly immunogenic and did not show any safety concerns in a GLP toxicology study. Further development of this vaccine is currently planned.

## Data Availability

Data are available upon request.
